# Real-world evidence to guide healthcare policies in oncology

**DOI:** 10.18632/oncotarget.27077

**Published:** 2019-07-16

**Authors:** Marco Donia, Steen Werner Hansen, Inge Marie Svane

**Affiliations:** ^1^ National Center for Cancer Immune Therapy (CCIT-DK), Department of Oncology, Herlev, Denmark; ^2^ Head Office, Herlev and Gentofte Hospital, Herlev, Denmark

**Keywords:** real-world evidence, real-world data, clinical trial eligibility

## Abstract

Randomized controlled clinical trials (RCTs) in oncology enroll patients who meet strict protocol-specified criteria. Many of these criteria overlap across multiple RCTs. A vast proportion of patients with metastatic cancer do not meet such criteria. Hence, patient populations encountered in clinical practice are essentially different from RCT-populations, questioning the representativeness of these trials. A real-world evidence approach, using data from clinical practice, is increasingly employed to complement the information on drug safety and efficacy obtained from traditional clinical trials.

## INTRODUCTION

The application of novel treatments to the broad real-world population of patients with a certain cancer disease often results in poorer outcomes than expected from pivotal clinical trials. In extreme cases, evidence from randomized controlled trials (RCT) [1, 2] appeared not to be applicable to the global real-world population [3]. The differences in outcome between RCT- and real-world population-based oncology studies are often believed to emerge from significant differences in the baseline characteristics of study participants. Strict enrolment criteria prevent a majority of RCTs from representing a sizable proportion of real-world patients, ranging from 35% to 70% in non-small cell lung- [4], renal- [5] and colorectal-cancer [6], and melanoma [7].

Given these important differences between RCT and real-world populations, the recent approval of numerous new treatments—with their associated high monetary costs and risks of toxicities—have led to a quickly increasing interest in the real-world evidence of the efficacy and safety of such therapies. Currently, a growing number of regulatory bodies, professional societies, and patient organizations support the use of real-world evidence for post-approval recommendations, health technology assessment, reimbursement policies and development of treatment guidelines. Currently, the broadest real-world evidence can be obtained from geographically defined whole population-based studies. These conditions may be satisfied by whole-population retrospective studies conducted within entire countries or regions with proven quality of data registration, such as those that are commonly carried out in Denmark [8].

**Figure 1:**
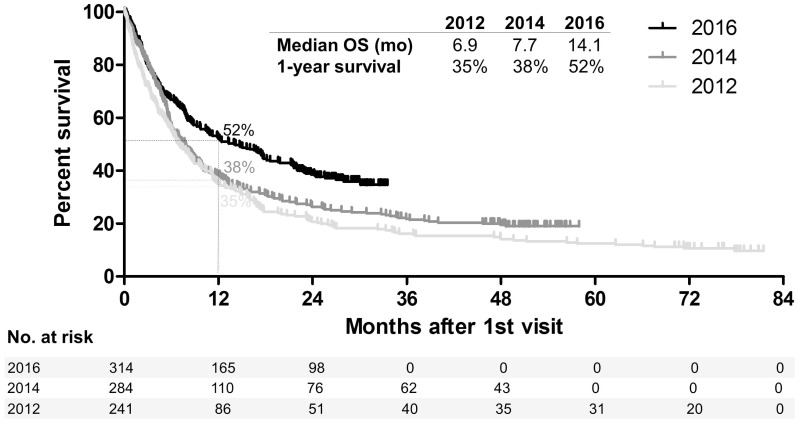
Survival of patients diagnosed with metastatic melanoma in the pre-modern era (2012), early-modern era (2014) and modern era (2016). Kaplan–Meier plot showing the survival of all patients diagnosed with melanoma, not amenable to surgery or other local treatment, in the whole population of Denmark in the calendar years 2012, 2014 and 2016. mo: months.

We have recently analyzed the differences in real-world outcome of all patients with metastatic melanoma diagnosed across Denmark in the pre-modern era (calendar year 2012, when BRAF-inhibitors but not first line immune checkpoint inhibitors where available), early-modern era (2014, first-line anti-CTLA-4 available) and modern era (2016, first line anti-PD-1 and MEK-inhibitors available) [9]. Despite similar baseline characteristics (data not shown), the survival outcome of the global metastatic melanoma population was significantly improved in 2016 versus 2014 (hazard ratio, HR for death 0.73, 95% CI 0.60–0.88; *p* = 0.0013) or 2012 (HR 0.61, 95% CI 0.50–0.75; *p* > 0.0001), with no major differences in 2014 versus 2012 (HR 0.85, 95% CI 0.70–1.03; *p* = 0.0935) (Figure 1). Importantly, we were able to discern a sub-group of real-world patients who had very similar baseline characteristics to patients enrolled in pivotal clinical-trials (“trial-like”), from a subgroup of patients that was not represented in such trials because they failed to meet one or more key criteria for enrolment (“trial-excluded”). As reported in our recent study [9], both groups had an improved outcome in 2016 versus 2014 or 2012. Hence, the introduction of novel treatments in 2016 led to a better survival outcome of the broad population of patients diagnosed with metastatic melanoma in the real-world. This largely confirms the results of phase III registration trials.

In conclusion, although RCTs are still the gold-standard practice to evaluate the efficacy (and safety) of a given treatment intervention versus the standard-of-care, real-world studies are critical for understanding whether the results from such RCTs are applicable to the broader population of patients affected by a certain disease. This is particularly true in oncology, where up to two-thirds of real-world patients [4–7] are not represented by current RCTs. As recently highlighted by a joint research statement of the American Society of Clinical Oncology (ASCO) and the Friends of Cancer Research [10], future RCTs should take these issues into account and broaden eligibility criteria to maximize the generalizability of results. Healthcare policies should be guided by data that are representative and generalizable to the global population of patients to whom a given treatment intervention offer is directed. To this end, we encourage healthcare regulators and reimbursement agencies to request such data. These data will be readily available, if future RCTs will broaden inclusion criteria. Nonetheless, it is likely that many future drug approvals will continue to be based on current types of unrepresentative RCTs; thus, we suggest that regulators demand a systematic follow up on expanded populations treated in the real-world on expanded populations in order to measure the true benefit of a certain treatment intervention. The recent European Medicine Agency’s Initiative for Patient Registries, created to optimize the continuing benefit-risk evaluation of medicinal products (already in use for CAR-T cel therapy post-authorisation follow up), is an important step forward in this direction.
